# Geniposide plus chlorogenic acid reverses non-alcoholic steatohepatitis *via* regulation of gut microbiota and bile acid signaling in a mouse model *in vivo*

**DOI:** 10.3389/fphar.2023.1148737

**Published:** 2023-04-03

**Authors:** Hongshan Li, Yingfei Xi, Xin Xin, Qin Feng, Yiyang Hu

**Affiliations:** ^1^ Institute of Liver Disease, Shuguang Hospital, Shanghai University of Traditional Chinese Medicine, Shanghai, China; ^2^ Liver Disease Department of Integrative Medicine, Ningbo No. 2 Hospital, Ningbo, Zhejiang, China; ^3^ Endocrine Department, Ningbo No. 2 Hospital, Ningbo, Zhejiang, China

**Keywords:** NASH, microbiota, bile acid, FXR, geniposide, chlorogenic acid

## Abstract

**Background:**

Geniposide and chlorogenic acid are the major active ingredients in Yinchenhao Decoction and are widely used as herbal medicines in Asia. This study further assessed their effects on improvement of non-alcoholic steatohepatitis (NASH) in a mouse model and explored the underlying molecular events *in vivo*.

**Methods:**

Male C57BL/6 and farnesoid X receptor knockout (FXR^−/−^) mice were used to establish the NASH model and were treated with or without geniposide, chlorogenic acid, obeticholic acid (OCA), and antibiotics for assessment of the serum and tissue levels of various biochemical parameters, bile acid, DNA sequencing of bacterial 16S amplicon, protein expression, and histology.

**Results:**

The data showed that the combination of geniposide and chlorogenic acid (GC) reduced the levels of blood and liver lipids, serum alanine aminotransferase (ALT), serum aspartate aminotransferase (AST), and the liver tissue index in NASH mice. In addition, GC treatment improved the intestinal microbial disorders in the NASH mice as well as the intestinal and serum bile acid metabolism. At the gene level, GC induced FXR signaling, i.e., increased the expression of *FXR*, small heterodimer partner (*SHP*), and bile salt export pump (*BSEP*) in liver tissues and fibroblast growth factor 15 (*FGF15*) expression in the ileal tissues of NASH mice. However, antibiotics (ampicillin, neomycin, vancomycin, and tinidazole) in drinking water (ADW) reversed the effect of GC on NASH and altered the gut microbiota in NASH mice *in vivo*. Furthermore, GC treatment failed to improve NASH in the FXR^−/−^ mouse NASH model *in vivo*, indicating that the effectiveness of GC treatment might be through FXR signaling activation.

**Conclusion:**

GC was able to alleviate NASH by improving the gut microbiome and activating FXR signaling; its effect was better than each individual agent alone.

## Introduction

Non-alcoholic fatty liver disease (NAFLD) is a commonly diagnosed liver disease in the world characterized by abnormal liver conditions, from liver steatosis and steatohepatitis to cirrhosis and eventually hepatocellular carcinoma (HCC) (Kumar et al., 2020). NAFLD mainly consists of two types, i.e., non-alcoholic fatty liver and non-alcoholic steatohepatitis (NASH) (Pierantonelli and Svegliati-Baroni, 2019). To date, about 25% of the worldwide population has been diagnosed with NAFLD, leading to increased numbers of NAFLD-associated cirrhosis and HCC (Araújo et al., 2018; Younossi et al., 2019; Paik et al., 2020). The risk factors of NAFLD include a diet high in fructose and an older age (Araújo et al., 2018; Younossi et al., 2018). In addition, those with NAFLD also have an increased risk of developing type 2 diabetes mellitus, cardiovascular disease, or chronic kidney disease (Byrne and Targher, 2015). The primary characteristic of NAFLD is lipid accumulation in the liver, usually as triglycerides (TGs), although the molecular mechanisms by which TGs lead to liver dysfunction remain to be defined (Marjot et al., 2020). Currently, various international practice guidelines are available to prevent and manage NAFLD, such as those provided by the American Association for the Study of Liver Diseases, the American Association of Clinical Endocrinology, the National Institute for Health and Care Excellence in England, the European Association for the Study of the Liver, and the Asia-Pacific Working Party on NAFLD organizations [European Association for the Study of the Liver (EASL) et al., 2016; Garvey et al., 2016; Glen et al., 2016; Chalasani et al., 2018; Chitturi et al., 2018; Wong et al., 2018]; however, effective drugs for the treatment or reversal of a simple fatty liver and NASH are still needed (Cai et al., 2019). Nevertheless, changes in non-healthy lifestyles and exercise have been shown to prevent or improve the disease (Hung and Bodenheimer, 2018), although a previous study has reported that these interventions might not completely block the progression of NAFLD to HCC (Lange et al., 2021). Thus, further investigation and identification of novel strategies or agents are urgently needed for the effective control of NAFLD progression in the clinical setting.

Geniposide is a bioactive iridoid glycoside that is present in various medicinal herbs, like *Gardenia jasminoides* (Zhou et al., 2019), and possesses a variety of activities, including neuroprotective, antidiabetic, hepatoprotective, anti-inflammatory, antioxidant, immune-regulatory, and antitumoral activities (Liu et al., 2015; Shan et al., 2017; Wang et al., 2017; Li et al., 2019). Meanwhile, chlorogenic acid is less investigated, and the available literature indicates that it can attenuate kidney fibrosis (Yunus et al., 2020), stabilize the immune system (Kzhyshkowska, 2022), and protect against the development of type 2 diabetes (Williamson, 2020). Both geniposide and chlorogenic acid are found in traditional Chinese herbs, such as Zhizi (Gardenia fruit) and Yinchen (Herba Artemisiae Scopariae), respectively. The Yinchenhao Decoction contains both and is widely prescribed in Asian countries (like China and Japan) to prevent and treat alcoholic liver disease, acute pancreatitis, and cholestasis (Xiang et al., 2016; Yan et al., 2017; Zhu et al., 2021). Our previous study has revealed that the combination of geniposide and chlorogenic acid (GC) improves and reduces high-fat diet (HFD)-induced NASH, improves intestinal barrier function (Peng et al., 2018), and downregulates the expression of stearoyl-CoA desaturase (Δ-9-desaturase) (SCD1) in NAFLD mice (Chen et al., 2021).

The present study further assessed the effectiveness of geniposide, chlorogenic acid, or their combination on improvement of NASH in a mouse model and then explored the underlying molecular events *in vivo*. We expect that our findings will provide novel information regarding their use in the control of NASH and the molecular events underlying their action against NASH.

## Materials and methods

### Establishment and treatment of a mouse model of NAFLD *in vivo*

The animal protocols used in this study were all approved by the Institutional Animal Care and Use Committee (IACUC) of Shanghai University of Traditional Chinese Medicine (Shanghai, China; approval #PZSHUTCM190531009) and followed the Guidelines for the Care and Use of Laboratory Animals provided by the Chinese Council on Animal Research.

For intervention of the model mice with geniposide, chlorogenic acid, or their combination, we purchased 50 specific pathogen-free (SPF), male C57BL/6 mice weighing 16–20 g from the Nanjing Biomedical Research Institute of Nanjing University [license number: SCXK (Su) 2015-0001] and housed them at the Experimental Animal Center of Ningbo University. After in-house adaption for 1 week, the animals were randomly separated into normal diet (ND; *n* = 10) and HFD (*n* = 40) groups. The mice in the HFD group were fed with a HFD (Cat. #D12492i; Research Diets, New Brunswick, NJ, United States), containing 60% of energy from fat and 5.21 kcal/g, for 10 weeks. Meanwhile, the mice in the ND group were fed a normal diet (Cat. #D12450B; Research Diets), containing only 10% energy from fat and 3.84 kcal/g, for 10 weeks. At the beginning of the 11th week, the HFD mice were further randomly divided into the HFD control, HFD + geniposide, HFD + chlorogenic acid, and HFD + GC groups (*n* = 10 per group), according to their body weight gradient. These mice were intragastrically administered with geniposide (90 mg/kg daily), chlorogenic acid (1.34 mg/kg daily), or their combination, respectively, for 4 weeks. The dosages used were the same as those reported previously (Peng et al., 2018), while the control mice intragastrically received an equal volume of drinking water for 4 weeks. Geniposide (Cat. #171130; purity >98%) and chlorogenic acid (Cat. #171209; purity >98%) were obtained from Shanghai Winherb Medical Technology Co., Ltd. (Shanghai, China).

Furthermore, we tested antibiotic treatment in 40 SPF, male C57BL/6 mice weighing 16–20 g from Shanghai Silaike Laboratory Animal Co., Ltd. [license number: SCXK (Shanghai)-2017-0003]. The animal experiments were conducted in a barrier animal room of the Experimental Animal Center, Shanghai University of Traditional Chinese Medicine. We randomly divided these mice into the following three groups: ND (*n* = 8), HFD control (*n* = 16), and HFD + antibiotics in drinking water (ADW) (*n* = 16). The HFD mouse model was established in the same manner as described in the previous section. After that, the HFD + ADW group of mice was given ADW starting at the 11th week for 4 weeks. Next, all HFD mice were further divided into the control and GC treatment groups (*n* = 8 in each group) at the 15th week after model establishment and intragastrically administered with the corresponding drugs (see above for details) for additional 4 weeks. The ADW was prepared by adding 1 g of ampicillin sodium salt (Cat. #A9518; Sigma, St. Louis, MO, United States), 1 g of neomycin trisulfate (Cat. #N6386; Sigma), 500 mg of vancomycin (Cat. #HY-17362/CS0908; MedChemExpress, Monmouth Junction, NJ, United States), and 1 g of tinidazole (Cat. #HY-B0177/CS-2055; MedChemExpress) in 1 L of mouse drinking water, and the solution was thoroughly mixed. The ADW was replaced every other day, as described previously (Pathak et al., 2018). The animal protocol of this experiment was approved by the IACUC of Shanghai University of Traditional Chinese Medicine (Shanghai, China; approval #PZSHUTCM190531009) and followed the Chinese Council on Animal Research Guidelines.

In addition, we tested the effect of obeticholic acid (OCA) treatment by using 36 SPF, male C57BL/6 mice weighing 16–20 g from Shanghai Silaike Experiment Animal Co., Ltd. (Shanghai, China). The experiments were performed in a barrier animal room of the Experimental Animal Center of Shanghai University of Traditional Chinese Medicine. The animals were randomly divided into ND (*n* = 9) and HFD (*n* = 27) groups, and the HFD model was established accordingly. At the 11th week, the HFD mice were further randomly divided into the following three groups: HFD control, HFD + GC, and HFD + OCA (*n* = 9 per group) and treated for 4 weeks according to a method described previously (Li et al., 2020). OCA was intragastrically administered at a dose of 10 mg/kg weight/day, according to a previous study (Li et al., 2020). The control mice were intragastrically administered an equal amount of drinking water for 4 weeks.

Furthermore, we purchased 23 male farnesoid X receptor (FXR) knockout (FXR^−/−^) mice weighing 16–20 g from Shanghai Model Organisms Biotechnology Co., Ltd. The breeder mice were originally from Jackson Laboratory (Bar Harbor, ME, United States). The experiments were performed in a barrier animal room of Shanghai University of Traditional Chinese Medicine Experimental Animal Center. In brief, these FXR^−/−^ mice were randomly divided into the FXR^−/−^ regular diet (FXR^−/−^ ND; *n* = 6) and FXR^−/−^ HFD (*n* = 17) groups for establishment of the non-alcoholic steatohepatitis model. After the 10th week, the FXR^−/−^ HFD animals were randomly separated into FXR^−/−^ HFD control, FXR^−/−^ HFD + GC, and FXR^−/−^ HFD + OCA groups, according to the body weight gradient, and treated accordingly for 4 weeks. The FXR^−/−^ ND and FXR^−/−^ HFD mice intragastrically received an equal amount of drinking water for 4 weeks.

### Specimen collection

After completion of each mouse experiment, the animals were first fasted for 12 h and then anaesthetized intraperitoneally with 3% sodium pentobarbital (3 mL/kg). Next, we opened the abdominal cavity along the midline to collect blood samples from the inferior vena cava. The blood samples were stored at 4°C for 4 h and then centrifuged for 15 min at 3,500 rpm in a clinical centrifuge. The serum samples were collected into new Eppendorf tubes and stored at −70°C. Meanwhile, we resected two small pieces of liver tissue (approximately 0.5 cm × 0.5 cm × 0.3 cm) from each mouse, one for formalin fixation and paraffin embedding, and another one for quickly freezing in liquid nitrogen and storage at −70°C. We also took the small intestine, colon, upper colon, and lower ileum and also placed them into Eppendorf tubes, respectively; the samples were quickly frozen in liquid nitrogen and stored at −70°C for future experiments. The liver index was calculated according to the following formula: wet liver weight/body weight × 100%.

### Assays of biochemical parameters in serum samples and liver tissues

The fresh mouse liver tissues were homogenized and processed for measurement of TG levels, as described previously (Li et al., 2020). The TG kit was provided by Nanjing Jiancheng Bioengineering Institute (Cat. #F001-1-1) and used according to their instructions.

The serum alanine aminotransferase (ALT) and aspartate aminotransferase (AST) levels were assayed using the ALT (Cat. #C009-2-1) and AST (Cat. #C010-2-1) testing kits from The Jiancheng Bioengineering Institute (Nanjing, China), according to their protocols. The fasting blood glucose (FBG) levels were assessed using the glucose oxidase kit from The Jiancheng Institute Bioengineering Institute (Cat. #F006-1-1), according to the protocol provided by the manufacturer.

The serum TG, total cholesterol (TC), low-density lipoprotein cholesterol (LDL-C), and high-density lipoprotein cholesterol (HDL-C) levels were assayed using the TG reagent kit (Batch number #201804007; Zhejiang Dongou Diagnostic Products Co., Ltd.), TC kit (Cat. #A111-1-1), LDL-C kit (Cat. #A113-1-1), and HDL-C kit (Cat. #A112-1-1; all from The Jiancheng Bioengineering Institute), according to their protocols.

The serum level of fasting insulin (FINS) was measured using a mouse FINS testing kit from Crystal Chem (Batch number #18MAUMI477A; Cat. #90080), according to a previous study (Li et al., 2020) and the manufacturer’s manual. After that, The Homeostatic Model Assessment of Insulin Resistance (HOMA-IR) was calculated using the HOMA methods, as described previously (Matthews et al., 1985).

### Hematoxylin and eosin (H&E) staining

To assess changes in liver tissue morphology, steatosis, and inflammation, 4-µm-thick tissue sections were prepared, stained with H&E, and then reviewed, scored, and photographed under a fluorescence microscope (LEICA 37XB, Wetzlar, Germany) by a liver pathologist in a data-blinded fashion. The NAFLD activity score (NAS) of the liver tissues was computed according to a previous study (Kleiner et al., 2005), i.e., steatosis (<5%, 0; 5%–33%, 1; 34%–66%, 2; >66%, 3), intralobular inflammation (none, 0; <2 lesions under a ×200 microscopic field, 1; 2–3 lesions/200 × field, 2; ≥4 lesions/200 × field, 3), and ballooning degeneration (none, 0; rare, 1; more common, 2).

### Oil red O staining

To assess changes in fatty acid accumulation in the liver tissues, 4-µm-thick frozen sections were prepared, stained with oil red O stain, and then reviewed, scored, and photographed under a Reka 37XB fluorescence microscope by a liver pathologist in data-blinded fashion.

### Liquid chromatography-mass spectrometry (LC-MS) to determine the bile acid levels in serum samples and the colon contents

A 20-µL aliquot of serum sample from each mouse was placed into a 96-well cell culture plate and mixed with 180 μL of acetonitrile/methanol (8:2) solution supplemented with 10 internal standards by placing the plate on a laboratory shake at a speed of 1,500 rpm at 10°C for 20 min to extract the metabolites. Next, the plate was centrifuged, and the supernatants were transferred to Eppendorf tubes for lyophilization using a freeze dryer (Labconco, Kansas City, MO, United States). The resulting powders were dissolved in a mixture of 1:1 (v/v) acetonitrile/methanol (80/20, v/v) and ddH_2_O containing lyophilized calibrator and then centrifuged at 13,500 rpm and 4°C for 20 min. Finally, 5 µL of each supernatant was added to a 96-well plate for LC-MS analysis of the bile acid levels.

Next, 10 mg of mouse colon contents was accurately weighed, placed in an Eppendorf tube containing 25 mg of beads and 20 µL of ddH_2_O, mixed well, and homogenized. After that, the samples were mixed with 180 µL of acetonitrile/methanol (8:2) solution supplemented with the 10 internal standards and centrifuged at 13,500 rpm and 4°C for 20 min in a Beckman Coulter Microfuge (Indianapolis, IN, United States). The supernatants were then added to a 96-well plate for lyophilization using a freeze dryer (Labconco). Next, the dried sample powders were mixed in a 1:1 ratio (v/v) of acetonitrile/methanol (80/20, v/v) and ddH_2_O containing lyophilized calibrator and then centrifuged at 13,500 rpm and 4°C for 20 min in a Beckman Coulter Microfuge; 5 µL of each sample was added to a 96-well plate for LC-MS analysis of the bile acid levels. Specifically, an ultrahigh-performance liquid chromatography-tandem mass spectrometry system (ACQUITY UPLC Xevo TQ-S, Waters Corp., Milford, MA, United States) was used to quantitatively analyze the bile acid levels, and all procedures were conducted by Shanghai Metabo-Profile Biotechnology (China).

### PCR and DNA sequencing of bacterial 16S amplicon

Genomic DNA was extracted from the fresh mouse colon contents with an EZNA^®^ Soil DNA kit (Omega Bio-Tek, Norcross, GA, United States), and the DNA purity and concentrations were estimated and assessed by 1% agarose gel electrophoresis. Next, these DNA samples were amplified for the V3–V4 variable region of the 16S rRNA gene using the 338F (5′-ACT​CCT​ACG​GGA​GGC​AGC​AG-3′) and 806R primers (5′-GGACTACHVGGGTWTCTAAT-3′). Each PCR contained 4 μL of 5× FastPfu buffer, 2 μL of 2.5 mM dNTPs, 0.8 μL of each forward and reverse primer, 0.4 μL of FastPfu polymerase, and 10 ng of template DNA in a total volume of 20 μL. The PCR program was initial denaturation at 95°C for 3 min and then 27 cycles of denaturation at 95°C for 30 s, annealing at 55°C for 30 s, and extension at 72°C for 30 s, and a final extension at 72°C for 10 min. The PCR products were kept at 4°C. The experiments were performed in triplicate and repeated at least once.

Next, these PCR products were subjected to Illumina MiSeq sequencing, i.e., the PCR products of each sample were separated on a 2% agarose gel, and the target products were further cleaned with an AxyPrep DNA Gel Extraction Kit (Axygen Biosciences, Union City, CA, United States) and inspected by 2% agarose gel electrophoresis. Subsequently, the purified PCR product was analyzed using a Quantus™ Fluorometer (Promega, Madison, WI, United States) for construction of the DNA library with a NEXTFLEX Rapid DNA-Seq Kit, according to the following procedures: 1) adaptor ligation; 2) magnetic bead screening to remove self-ligated fragments of the adaptor; 3) PCR amplification of the enriched library template; and 4) use of magnetic beads to recover the PCR products and to generate the final DNA library for DNA sequencing using the MiSeq PE300 platform of Illumina (performed by The Majorbio Biopharmaceutical Technology, Shanghai, China). The DNA sequencing data were subjected to a quality-control step using Trimmomatic software, and the assembly was determined using FLASH software. After that, we utilized UPARSE software (version 7.1 http://drive5.com/uparse/) to analyze the operational taxonomic unit clustering of the sequences using 97% similarity as a cut-off value to exclude chimaeras and then the RDP classifier (http://rdp.cme.msu.edu/) to classify the species of each DNA sequence. The assessment threshold was set to 70% based on the Silva database data (SSU128).

### Western blot

Total cellular protein was extracted from the mouse liver and ileal samples using radioimmunoprecipitation assay buffer (Cat. #P0013B; Beyotime, Shanghai, China) supplemented with the proteinase inhibitors (Cat. #P1045-1; Beyotime) and subjected to the bicinchoninic acid protein assay kit (Lot number: TI269557; Thermo Fisher, Waltham, MA, United States) to determine the concentration, according to a previous study (Li et al., 2020). The BeyoECL Plus A solution (Cat. #P0018 M-1) and BeyoECL Plus B solution (Cat. #P0018 M-2) were purchased from Beyotime to assess the positive protein bands in the membranes. Protein separation by gel electrophoresis and Western blot analysis were performed according to a method described previously (Li et al., 2020).

The primary antibodies used were monoclonal anti-FXR (Cat. #A9033A; 1:1,000; Thermo Fisher), anti-small heterodimer partner (SHP) (Cat. #PA5-76632; 1:1,000; Thermo Fisher), anti-cytochrome P450 family 7 subfamily A member 1 (CYP7A1) (Cat. #ab65596; 1:1,000; Abcam, Cambridge, MA, United States) and polyclonal anti-bile salt export pump (BSEP) (Cat. #PA5-78690; 1:2000; Thermo Fisher), anti-sodium taurocholate cotransporting polypeptide (NTCP) (Cat. #PA5-80001; 1:2,000; Thermo Fisher), anti-fibroblast growth factor 15 (FGF15) (Cat. #ab229630; 1:1,000; Abcam), anti-fibroblast growth factor receptor 4 (FGFR4) (Cat. #ab119378; 1:500; Abcam), anti-Klotho beta (KLB) (Cat. #ab233416; 1:1,000; Abcam), anti-sterol regulatory element-binding protein 1 (Cat. #ab28481; 1:1,000; Abcam), anti-SCD1 (Cat. #ab19862; 1:1,000; Abcam), anti-fatty acid synthetase (Cat. #3189; 1:1,000; Cell Signaling Technology, Danvers, MA, United States), anti-carnitine palmitoyltransferase 1A antibody, (Cat. #ab128568; 1:1,000; Abcam), anti-lipoprotein lipase antibody (Cat. #ab21356; 1:1,000; Abcam). The rabbit polyclonal anti-glyceraldehyde 3-phosphate dehydrogenase antibody was from Proteintech Group, Inc. (Cat. #10494-1-AP; 1:1,000), while the goat anti-mouse IgG (Cat. #IH-0031) and goat anti-rabbit IgG (Cat. #IH-0011) were purchased from Beijing Dingguo Changsheng Biotechnology Co., Ltd.

### Statistical analysis

The data were summarized as the mean ± standard deviation and analyzed statistically with SPSS 16.0 software (SPSS, Chicago, IL, United States). The normally distributed data were analyzed using one-way analysis of variance, while the least-significant difference test was used to assess and compare the difference between two groups. However, the non-normally distributed data were assessed with the non-parametric test. A *p*-value < 0.05 was set as statistical significance. The gut microbiota data were assessed with the online Majorbio cloud platform (www.majorbio.com).

## Results

### GC reduces liver lipid and serum ALT and AST levels in a mouse NASH model

**FIGURE 1:**
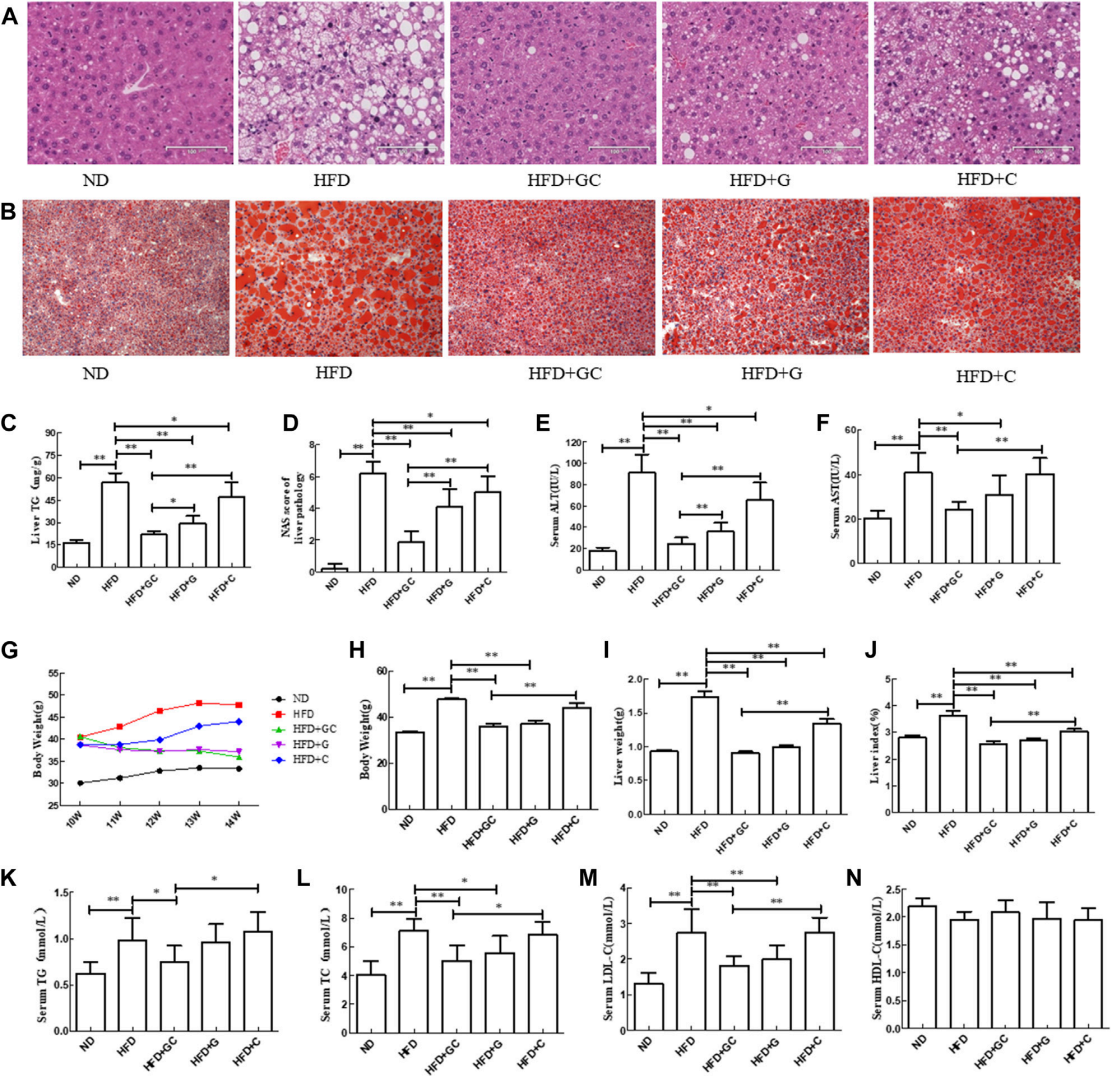
Changes in liver histology as well as biochemical indexes of liver and serum in a mouse NAFLD model with intervention of geniposide, chlorogenic acid, or their combination. **(A)** H&E staining, ×200. **(B)** Oil red O staining, ×200. **(C)** Liver TG level. **(D)** Liver NAS. **(E)** Serum ALT level. **(F)** Serum AST level. **(G)** Mouse body weight. **(H)** Mouse body weight. **(I)** Mouse wet liver weight. **(J)** Mouse liver index, wet liver weight/body weight × 100%. **(K)** Serum TG level. **(L)** Serum TC level. **(M)** Serum LDL-C level. **(N)** Serum HDL-C level. Abbreviations: ND, normal diet; HFD, high-fat diet; GC, geniposide and chlorogenic acid combination treatment; G, geniposide treatment; C, chlorogenic acid treatment; TG, triglyceride; ALT, alanine aminotransferase; AST, serum aspartate aminotransferase; NAFLD, non-alcoholic fatty liver disease; H&E, hematoxylin and eosin; NAS, NAFLD activity score. *p < 0.05 and **p < 0.01 (*n* = 10 per group).

First, we established a mouse NAFLD model *in vivo*, and our H&E staining data showed that the hepatocytes had obvious steatosis, accompanied by scattered inflammatory cell infiltration, and ballooning degeneration in the mouse liver tissues of the HFD model. A number of hepatocytes with steatosis were swollen and rounded, and there was accumulation of fat droplets in the cytoplasm. However, treatment of mice with geniposide, chlorogenic acid, or their combination showed significant effects on alleviation of this liver morphology, especially in the HFD + GC group of mice (Figure 1A).

Furthermore, the oil red O staining results showed that the liver tissue in the HFD mice exhibited obvious hepatic steatosis, with more large and round fat droplets in the cytoplasm compared with those of the liver tissues from ND mice. In contrast, the liver histology was significantly improved after treatment with geniposide, chlorogenic acid, or their combination, especially in the HFD + GC group of mice (Figure 1B). Moreover, the TG level and NAS were both much higher in the HFD liver tissues than in the ND ones (*p* < 0.01; Figures 1C, D). However, the TG level and NAS were significantly lower in the liver tissues of the treatment mice vs. those in the HFD control group (*p* < 0.01), and the effect was more obvious in the liver tissues of the combination-treated mice (Figures 1C, D). In addition, the serum ALT and AST levels were dramatically increased in the HFD mice vs. the ND mice (*p* < 0.01). In contrast, the serum ALT and AST levels in the GC-treated mice and the geniposide-treated mice as well as the serum ALT level in the chlorogenic acid-treated mice were all significantly lower vs. those of the HFD mice (*p* < 0.05). The combination treatment showed more obvious effects on reducing the serum ALT level than that of treatment with an individual drug (*p* < 0.01; Figures 1E, F).

### GC reduces the liver index and blood lipid levels in NASH mice

Next, we assessed the mouse body weight and found that during the experiment, the ND mouse body weight slowly increased, whereas the body weight in the model control and chlorogenic acid-treated model mice showed an increasing trend. In contrast, the body weights of the geniposide-treated and GC-treated mice showed a gradually increasing trend. When the experiment was finished, the HFD mouse body weight was significantly greater than that of the ND mice (*p* < 0.01). On the contrary, the body weights of the HFD + GC and HFD + geniposide mice were much less than that of the HFD mice (*p* < 0.01). The body weight of the HFD + GC mice was less than those of the HFD + chlorogenic acid mice (*p* < 0.01) and the HFD + geniposide mice, although the difference was not statistically significant for the latter group (*p* > 0.05; Figures 1G, H).

Furthermore, the HFD mouse wet liver weight and liver index were much greater than those of the ND mice (*p* < 0.01). However, the wet liver weight and liver index of the HFD + GC, HFD + geniposide, and HFD + chlorogenic acid mice were considerably less than those of the HFD mice (*p* < 0.01), with the most obvious difference in the HFD + GC mice (*p* < 0.01; Figures 1I, J). Moreover, the serum TG, TC, and LDL-C levels were significantly increased in the model control mice vs. those in the ND mice (*p* < 0.01), whereas their levels were decreased in the HFD + GC mice (*p* < 0.05). The serum TC and LDL-C levels were also lower in the HFD + geniposide mice (*p* < 0.05), although the *p*-value of the serum HDL-C level among the groups was not statistically significant (*p* > 0.05; Figures 1K–N).

### Antibiotic treatment reverses the effect of GC on NASH in a mouse model

**FIGURE 2:**
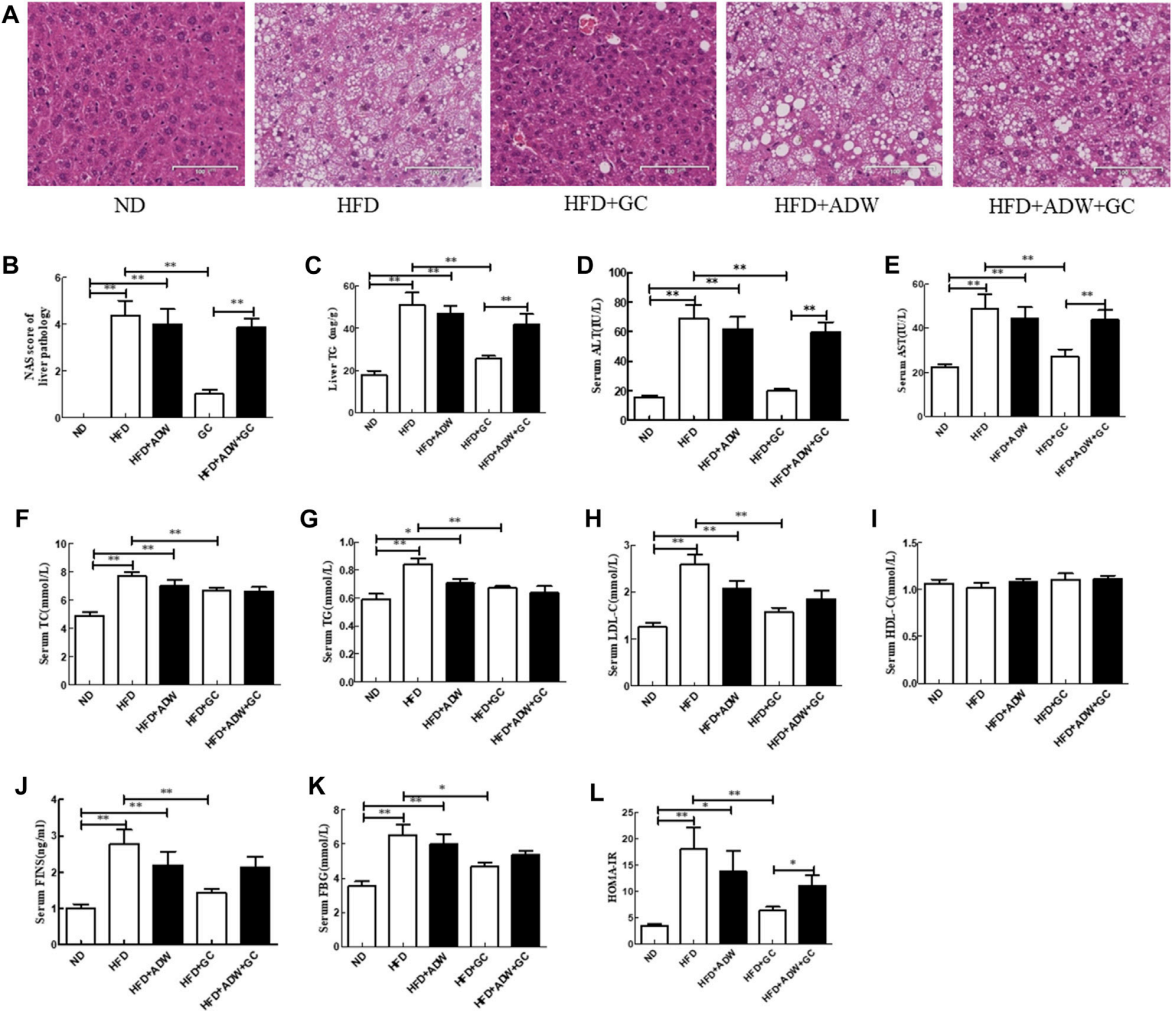
Changes in liver histology and biochemical indexes of liver and serum in a mouse NAFLD model and intervention with GC with or without ADW. **(A)** H&E staining, ×200. **(B)** Liver NAS. **(C)** Liver TG level. **(D)** Serum ALT level. **(E)** Serum AST level. **(F)** Serum TC level. **(G)** Serum TG level. **(H)** Serum LDL-C level. **(I)** Serum HDL-C level. **(J)** Serum FINS level. **(K)** Serum FBG level. **(L)** HOMA-IR. Abbreviations: NAFLD, non-alcoholic fatty liver disease; ND, normal diet; HFD, high-fat diet; ADW, antibiotic drinking water (containing ampicillin, neomycin, vancomycin, and tinidazole); GC, geniposide and chlorogenic acid combination treatment; NAS, NAFLD activity score; H&E, hematoxylin and eosin; TG, triglyceride; ALT, alanine aminotransferase; AST, serum aspartate aminotransferase; TC, total cholesterol; LDL-C, low-density lipoprotein cholesterol; HDL-C, high-density lipoprotein cholesterol; FINS, fasting insulin; FBG, fasting blood glucose; HOMA-IR, Homeostatic Model Assessment of Insulin Resistance. *p < 0.05 and **p < 0.01 (*n* = 8 per group).

To further assess the effect of the GC combination treatment on improving NASH in a mouse model *in vivo*, we added antibiotic treatment to the experimental mice. Our data showed that the liver structure in the HFD mice and the HFD + ADW mice exhibited obvious hepatic steatosis and accumulation of fatty acid vacuoles in the cytoplasm. In addition, a number of hepatocytes were extremely swollen and had scattered ballooning degeneration with infiltrated inflammatory cells. These changes were significantly improved in the HFD + GC mice, whereas there was no obvious improvement of liver histology in the HFD + ADW + GC mice (Figure 2A).

Furthermore, the TG level and NAS were much greater in the HFD mice and the HFD + ADW mice than in the ND mice (*p* < 0.01); however, the GC combination treatment significantly decreased the TG level and NAS vs. the model mice (*p* < 0.01). In contrast, ADW blocked the effects of geniposide and chlorogenic acid, i.e., the TG level and NAS showed no significant difference between the HFD + ADW mice and the HFD + ADW + GC mice (*p* > 0.05), and the NAS was much greater in the HFD + ADW + GC mice than in the HFD + GC mice (*p* < 0.01; Figures 2B, C).

In addition, the serum ALT, AST, TG, TC, and LDL-C levels were much greater in the HFD mice and the HFD + ADW mice than in the ND mice (*p* < 0.01 or *p* < 0.05), whereas their levels were much lower in the HFD + GC mice than in the model mice (*p* < 0.01). The serum levels of ALT, AST, TG, TC, and LDL-C showed no statistically significant changes between the HFD + ADW + GC mice and the HFD + ADW mice (*p* > 0.05), although the serum ALT and AST levels were lower in the HFD + GC mice (*p* < 0.01). However, statistical significance was not reached for serum HDL-C level among these mice (*p* > 0.05; Figures 2D–I). Moreover, the serum FINS level, FBG, and HOMA-IR were all much greater in the HFD mice and the HFD + ADW mice than in the ND mice (*p* < 0.05), whereas these levels were significantly decreased in the HFD + GC mice vs. those in the HFD mice (*p* < 0.05). However, these parameters were not significantly different between the HFD + ADW mice and the HFD + ADW + GC mice (*p* > 0.05), and the HOMA-IR was increased in the HFD + ADW + GC mice compared with the HFD + GC mice (*p* < 0.05; Figures 2J–L).

### Antibiotic treatment changes the gut microbiota in a mouse NASH model

**FIGURE 3:**
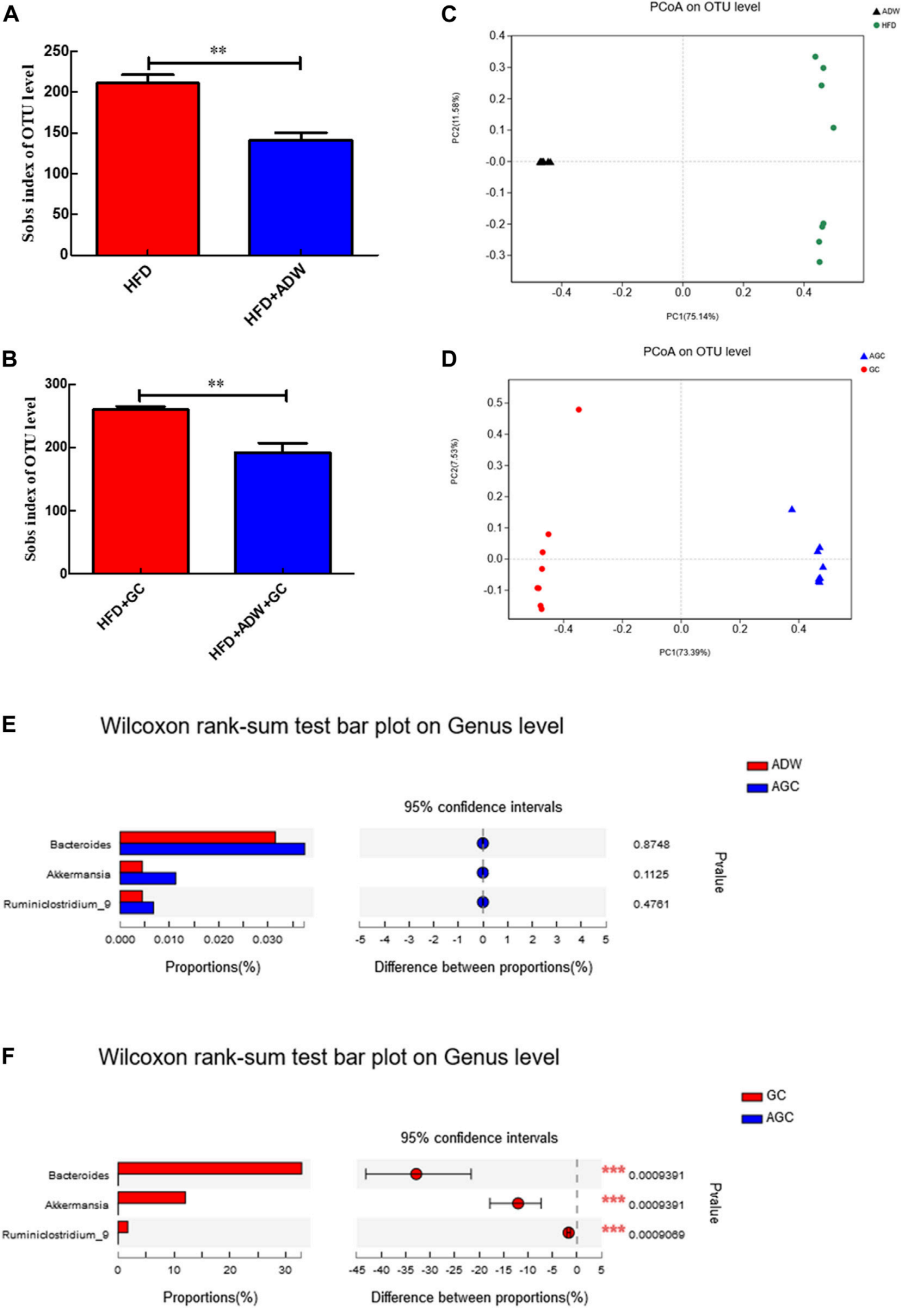
Changes in the gut microbiota and intervention with GC with or without ADW in a mouse NAFLD model. **(A, B)** Alpha diversit of the gut microbiota. **(C, D)** Beta diversity analyzed using PCoA of the gut microbiota in each labeled group. **(E)** Wilcoxon rank-sum test results of changes at the genus level between the ADW and AGC groups. **(F)** Wilcoxon rank-sum test results of changes at the genus level between the GC and AGC groups. Abbreviations: NAFLD, non-alcoholic fatty liver disease; ADW, antibiotic drinking water (containing ampicillin, neomycin, vancomycin, and tinidazole); GC, geniposide and chlorogenic acid combination treatment; ND, normal diet; HFD, high-fat diet; PCoA, principal coordinate analysis; OTU, operational taxonomic unit. **p < 0.01 (*n* = 8 per group).

We then assessed the alteration of the gut microbiota in NASH mice. Our data revealed that the alpha diversity of the gut microbiota was much lower in the HFD + ADW mice than in the HFD mice (*p* < 0.01) as well as in the HFD + ADW + GC mice than in the HFD + GC mice (*p* < 0.01; Figures 3A, B). Moreover, the principal coordinate analysis based on UniFrac speculated that the differences in the composition of the gut microbiota in the same mouse group were small and that differences in the composition of the gut microbiota between the HFD and HFD + ADW mice or between the HFD + GC and HFD + GC + ADW mice were greater (Figures 3C, D), indicating that antibiotic treatment altered the gut microbiota in NASH mice. Indeed, the abundance of *Bacteroides*, *Ruminiclostridium_9*, and *Akkermansia* showed no significant change in the ADW + GC mice vs. the ADW mice (*p* > 0.05), whereas they were much lower in the ADW + GC mice than in the HFD + GC mice (*p* < 0.01; Figures 3E, F).

### Lost effects of GC on the liver lipid level in *FXR*-knockout NASH mice

**FIGURE 4:**
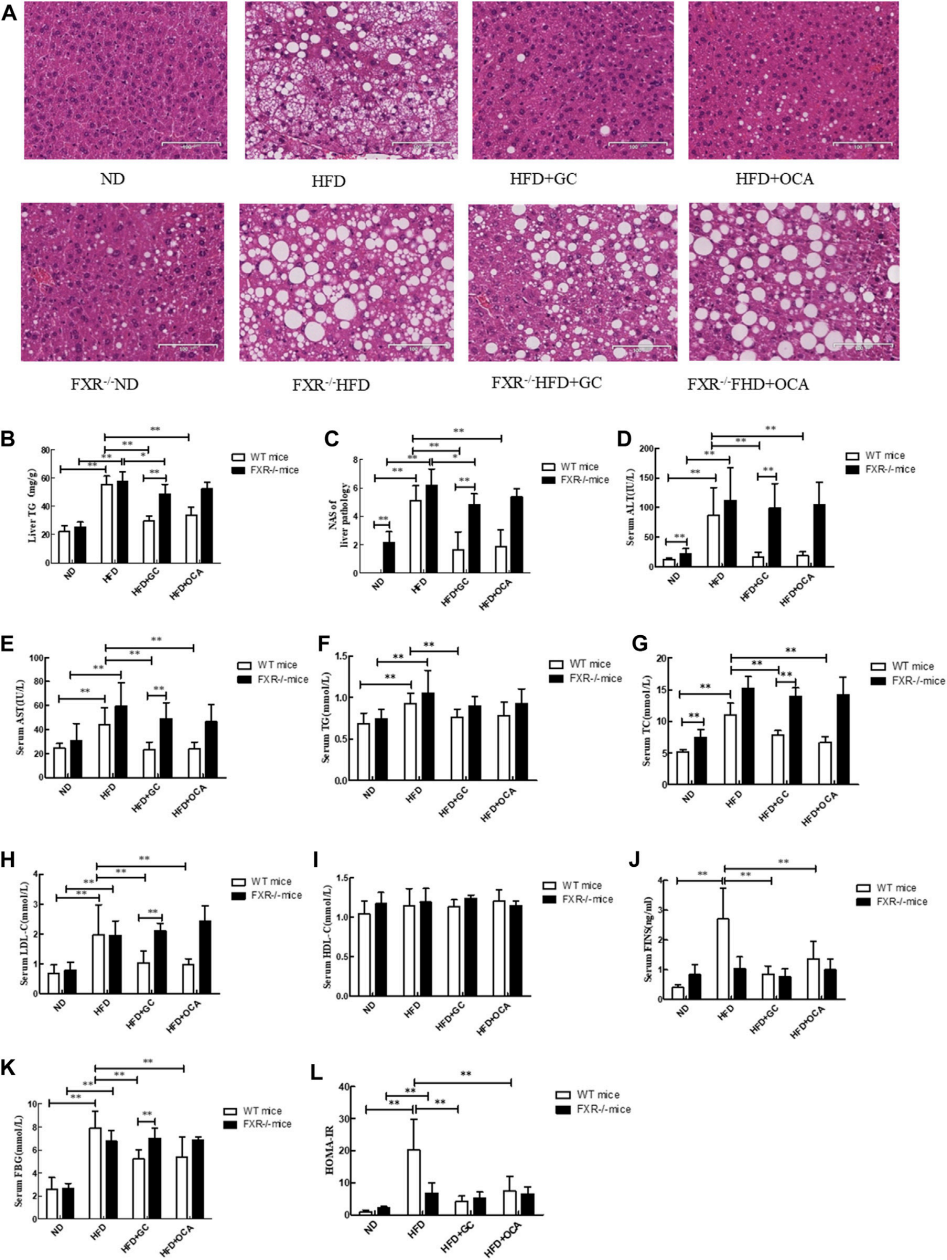
Changes in liver morphology, NAS and biochemical indexes with intervention of GC or OCA in wild-type and FXR^−/−^ mouse NAFLD models. **(A)** H&E staining, ×200. **(B)** Liver TG level. **(C)** Liver NAS. **(D)** Serum ALT level. **(E)** Serum AST level. **(F)** Serum TG level. **(G)** Serum TC level. **(H)** Serum LDL-C level. **(I)** Serum HDL-C level. **(J)** Serum FINS level. **(K)** Serum FBG level. **(L)** HOMA-IR. Abbreviations: NAFLD, non-alcoholic fatty liver disease; ND, normal diet; HFD, high-fat diet; GC, geniposide and chlorogenic acid combination treatment; OCA, obeticholic acid; TG, triglyceride; NAS, NAFLD activity score; ALT, alanine aminotransferase; AST, serum aspartate aminotransferase; TG, triglyceride; TC, total cholesterol; LDL-C, low-density lipoprotein cholesterol; HDL-C, high-density lipoprotein cholesterol; FINS, fasting insulin; FBG, fasting blood glucose; HOMA-IR, Homeostatic Model Assessment of Insulin Resistance; WT, wild type; FXR^−/−^, FXR knockout. *p < 0.05 and **p < 0.01 (*n* = 9 per group).

We also detected the effects of *FXR* knockout on regulating geniposide and chlorogenic acid activity against NASH formation in mice and established the NASH model in *FXR*-knockout mice. In the wild-type mice, the TG level and NAS in the liver tissues were much greater in the HFD mice than in the ND mice (*p* < 0.01), whereas these parameters became much lower in the HFD + GC mice and the HFD + OCA mice than in the HFD mice (*p* < 0.01), although statistical significance was not reached between the HFD + GC mice and the HFD + OCA mice (*p* > 0.05; Figures 4B, C). Moreover, the mouse liver tissues had obvious steatosis with a large number of fatty acid droplets in the cell cytoplasm, infiltration of inflammatory cells, and ballooning degeneration in the HFD mice, whereas the liver histology was significantly improved after GC and OCA treatment, i.e., in the HFD + GC and HFD + OCA mice (Figure 4A).

However, the TG level and NAS were both upregulated in the FXR^−/−^ HFD mice vs. the FXR^−/−^ ND mice (*p* < 0.01), whereas their levels were downregulated in the FXR^−/−^ HFD + GC mice vs. the FXR^−/−^ HFD mice (*p* < 0.05). In contrast, their levels were much greater in the FXR^−/−^ HFD + GC mice than in the HFD + GC mice (*p* < 0.01). Statistical significance was not reached for the liver tissue TG level between the FXR^−/−^ HFD + OCA mice and the HFD + OCA mice (*p* > 0.05; Figures 4B, C).

Furthermore, the liver tissue structure in the FXR^−/−^ HFD mice showed obvious steatosis, i.e., accumulation of fatty acid droplets in the hepatocyte cytoplasm, infiltration of inflammatory cells, and ballooning degeneration. In contrast, the GC treatment alleviated such changes in the liver histology in FXR^−/−^ mice, although OCA treatment did not (Figure 4A).

### Lost effects of GC on biochemical indicators in *FXR*-knockout NASH mice

Next, we assessed the changes in the biochemical indicators in *FXR*-knockout NASH mice. We first performed experiments on wild-type mice and found that the serum ALT and AST levels were much higher in the HFD mice than in the ND mice (*p* < 0.01), whereas their levels were dramatically downregulated in the HFD + GC and HFD + OCA mice vs. the HFD mice (*p* < 0.01), although statistical significance was not reached between the HFD + GC mice and the HFD + OCA mice (*p* > 0.05; Figures 4D, E). Moreover, the serum TC, TG, and LDL-C levels were also higher in the HFD mice vs. the ND mice (*p* < 0.01), whereas the serum TC, TG, and TG levels in the HFD + GC mice and the serum TC and LDL-C levels in the HFD + OCA mice were all much lower than in the HFD mice (*p* < 0.01), although statistical significance among the serum TC, TG, and LDL-C levels was not reached between the HFD + GC mice and HFD + OCA mice (*p* > 0.05) or for the HDL-C levels among the mice (*p* > 0.05; Figures 4F–I). The serum levels of FINS and FBG as well as HOMA-IR were also increased in the HFD mice compared with those of the ND mice (*p* < 0.01), whereas these parameters were significantly downregulated in the HFD + GC and HFD + OCA mice vs. the HFD mice (*p* < 0.01). The serum FINS level and HOMA-IR were much lower in the HFD + GC mice vs. the HFD + OCA mice (*p* < 0.05) (Figures 4J–L).

However, in the FXR^−/−^ mice, the serum ALT, AST, TG, TC, and LDL-C levels were much greater in the FXR^−/−^ HFD mice than in the FXR^−/−^ ND mice (*p* < 0.01), but treatment did not improve these parameters in the FXR^−/−^ HFD + GC mice or the FXR^−/−^ HFD + OCA mice vs. the FXR^−/−^ HFD mice (*p* > 0.05; Figures 4D–H). Moreover, the serum FBG level and HOMA-IR were dramatically higher in the FXR^−/−^ HFD mice than in the FXR^−/−^ ND mice (*p* < 0.01), whereas these parameters were not improved after treatment, including in the FXR^−/−^ HFD + GC mice or the FXR^−/−^ HFD + OCA mice vs. the FXR^−/−^ HFD mice (*p* > 0.05; Figures 4J–L).

### GC improves intestinal microbial disorders in NASH mice

**FIGURE 5:**
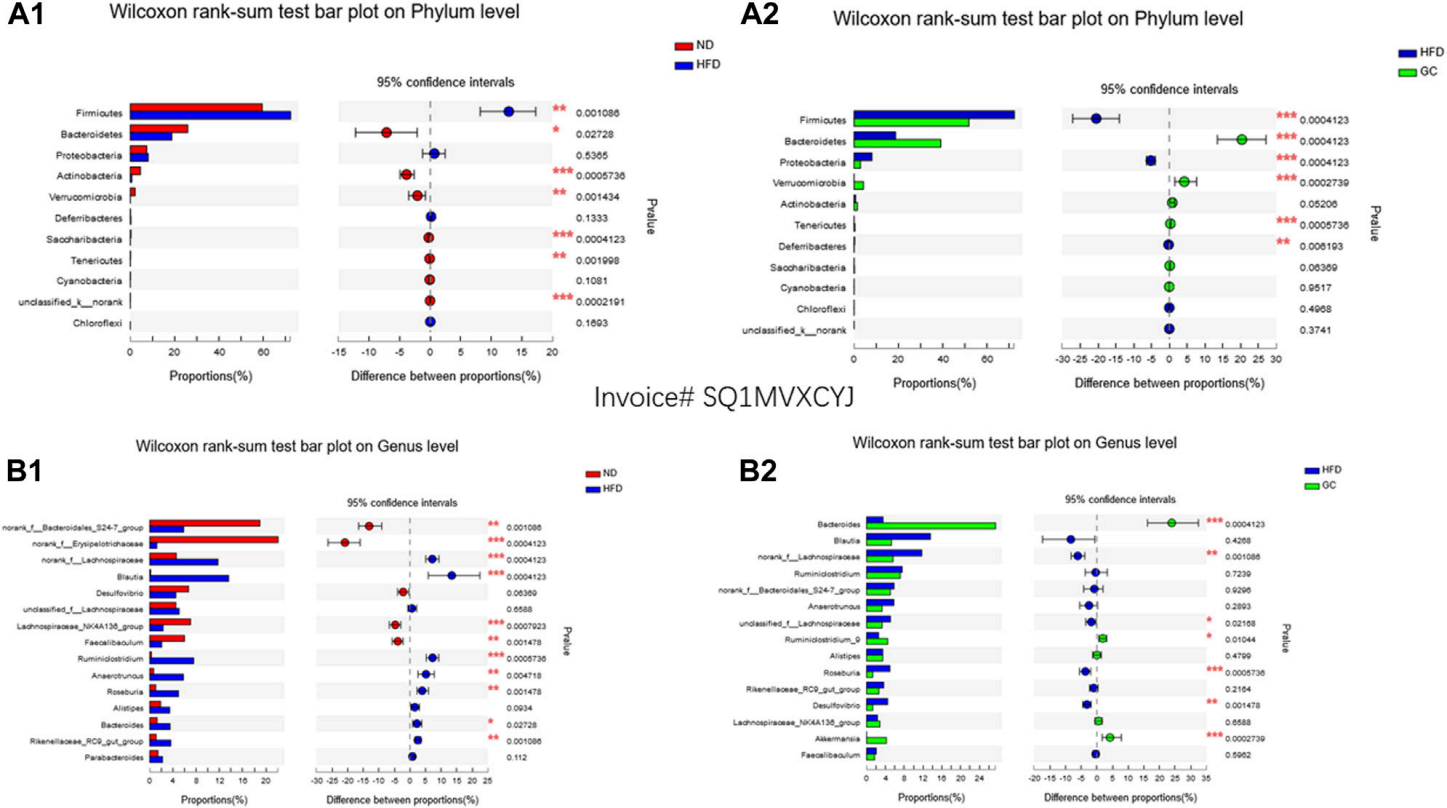
Changes in the gut microbiota and intervention with GC in a mouse NAFLD model. The gut microbiota data were analyzed by using the online Majorbio cloud platform (www.majorbio.com). **(A1)** Wilcoxon rank-sum test of changes at the phylum level. The data show the changes in the gut microbiota of mice in the HFD and ND groups at the phylum level. **(A2)** Wilcoxon rank-sum test of changes at the phylum level. The data show the changes in the gut microbiota of mice in the HFD + GC and HFD groups at the phylum level. **(B1)** Wilcoxon rank-sum test of changes at the genus level. The data show the changes in the gut microbiota of mice in the HFD and ND groups at the genus level. **(B2)** Wilcoxon rank-sum test of changes at the genus level. The data show the changes in the gut microbiota of mice in the HFD + GC and HFD groups at the genus level. Abbreviations: NAFLD, non-alcoholic fatty liver disease; ND, normal diet; HFD, high-fat diet; GC, HFD + geniposide and chlorogenic acid combination treatment. *p < 0.05, **p < 0.01, and ***p < 0.001 (*n* = 9 per group).

At the phylum level, the abundance of intestinal Firmicutes was much higher (*p* < 0.01), whereas the abundance of Bacteroidetes, Actinobacteria, Verrucomicrobia, Tenericutes, and Saccharibacteria was significantly lower in the HFD mice than in the ND mice (*p* < 0.05; Figure 5A1). However, the abundance of intestinal Firmicutes, Proteobacteria, and Deferribacteres was even lower (*p* < 0.01), but the abundance of Bacteroidetes, Verrucomicrobia, and Tenericutes was much increased in the HFD + GC mice compared with those in the HFD mice (*p* < 0.01; Figure 5A2).

At the genus level, the abundance of intestinal *Blautia*, *norank _f_Lachnospiraceae*, *Ruminiclostridium*, *Anaerotruncu*, *Bacteroides*, *Rikenellaceae*_RC9_gut_group, and *Roseburia* was much higher (*p* < 0.05), whereas the abundance of *norank _f_f_Lachnospiraceae*, *norank _f_Erysipelotrichaceae*, *Lachnospiraceae*_NK4A136_group, and *Faecalibaculum* was much lower in the HFD mice than in the ND mice (*p* < 0.01; Figure 5B1). However, the abundance of intestinal *Bacteroides*, *Ruminiclostridium*_9, and *Akkermansia* was upregulated (*p* < 0.05), and the abundance of unclassified_f_*Lachnospiraceae*, *norank_f_Lachnospiraceae*, *Roseburia*, and *Desulfovibrio* was downregulated in the HFD + GC mice vs. the HFD mice (*p* < 0.05; Figure 5B2).

### GC improves bile acid metabolism in NASH mice

**FIGURE 6:**
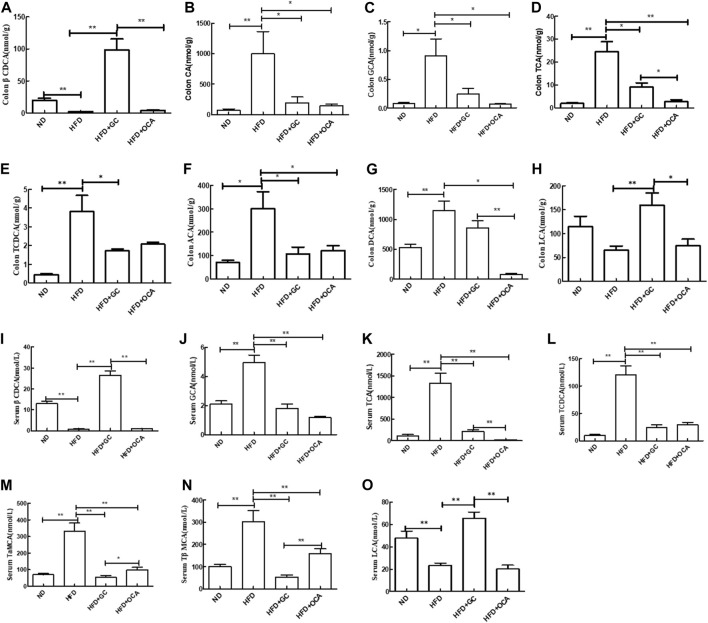
Changes in the bile acid levels in the colon contents and serum with intervention of GC or OCA in a mouse NAFLD model. **(A)** βCDCA in colon content. **(B)** CA in colon content. **(C)** GCA in colon content. **(D)** TCA in colon content. **(E)** TCDCA in colon content. **(F)** ACA in colon content. **(G)** DCA in colon content. **(H)** LCA in colon content. **(I)** Serum βCDCA level. **(J)** Serum GCA level. **(K)** Serum TCA level. **(L)** Serum TCDCA level. **(M)** Serum TaMCA level. **(N)** Serum TβMCA level. **(O)** Serum LCA level. Abbreviations: NAFLD, non-alcoholic fatty liver disease; DCA, deoxycholic acid; GCA, glycocholic acid; βCDCA, 3β-chenodeoxycholic acid; TCA, taurocholic acid; TCDCA, taurochenodeoxycholic acid; CA, cholic acid; ACA, allocholic acid; TβMCA, tauro β-muricholic acid; TαMCA, tauro α-muricholic acid; LCA, lithocholic acid. ND, normal diet; HFD, high-fat diet; GC, geniposide and chlorogenic acid combination treatment; OCA, obeticholic acid. *p < 0.05 and **p < 0.01 (*n* = 5 per group).

The levels of cholic acid (CA), taurocholic acid (TCA), glycocholic acid (GCA), taurochenodeoxycholic acid (TCDCA), allo-cholic acid (ACA), and deoxycholic acid (DCA) were all much higher (*p* < 0.05), whereas the 3β-chenodeoxycholic acid (βCDCA) level was significantly reduced in the intestinal tissues of the HFD mice vs. those in the ND mice (*p* < 0.01). The CA, TCA, GCA, TCDCA, and ACA levels were all downregulated in the HFD + GC mice vs. the HFD mice, and the CA, TCA, GCA, ACA, and DCA levels were also reduced in the HFD + OCA group compared to those of the HFD mice (*p* < 0.05), whereas the levels of βCDCA and lithocholic acid (LCA) were significantly increased in the HFD + GC mice (*p* < 0.01). In addition, the levels of DCA, TCA, LCA, and βCDCA were much higher in the HFD + GC mice than in the HFD + OCA mice (*p* < 0.05; Figures 6A–H).

Furthermore, the serum levels of TCA, GCA, TCDCA, tauro-alpha-muricholic acid (TaMCA), and tauro-β-muricholic acid (TβMCA) were much higher (*p* < 0.01), whereas the βCDCA and LCA levels were much lower in the HFD mice than in the ND mice (*p* < 0.01). In contrast, the serum levels of TCA, GCA, TCDCA, TaMCA, and TβMCA were much lower (*p* < 0.01) in the HFD + GC mice and HFD + OCA mice, whereas the serum βCDCA and LCA levels were much higher in the HFD + GC mice than in the HFD mice (*p* < 0.01). Moreover, the serum levels of TaMCA and TβMCA were much lower (*p* < 0.05), whereas the serum levels of TCA, LCA, and βCDCA were higher in the HFD + GC mice vs. the HFD + OCA mice (*p* < 0.01, Figures 6I–O).

### GC improves FXR signaling, but the effect is lost in *FXR*-knockout mice

**FIGURE 7:**
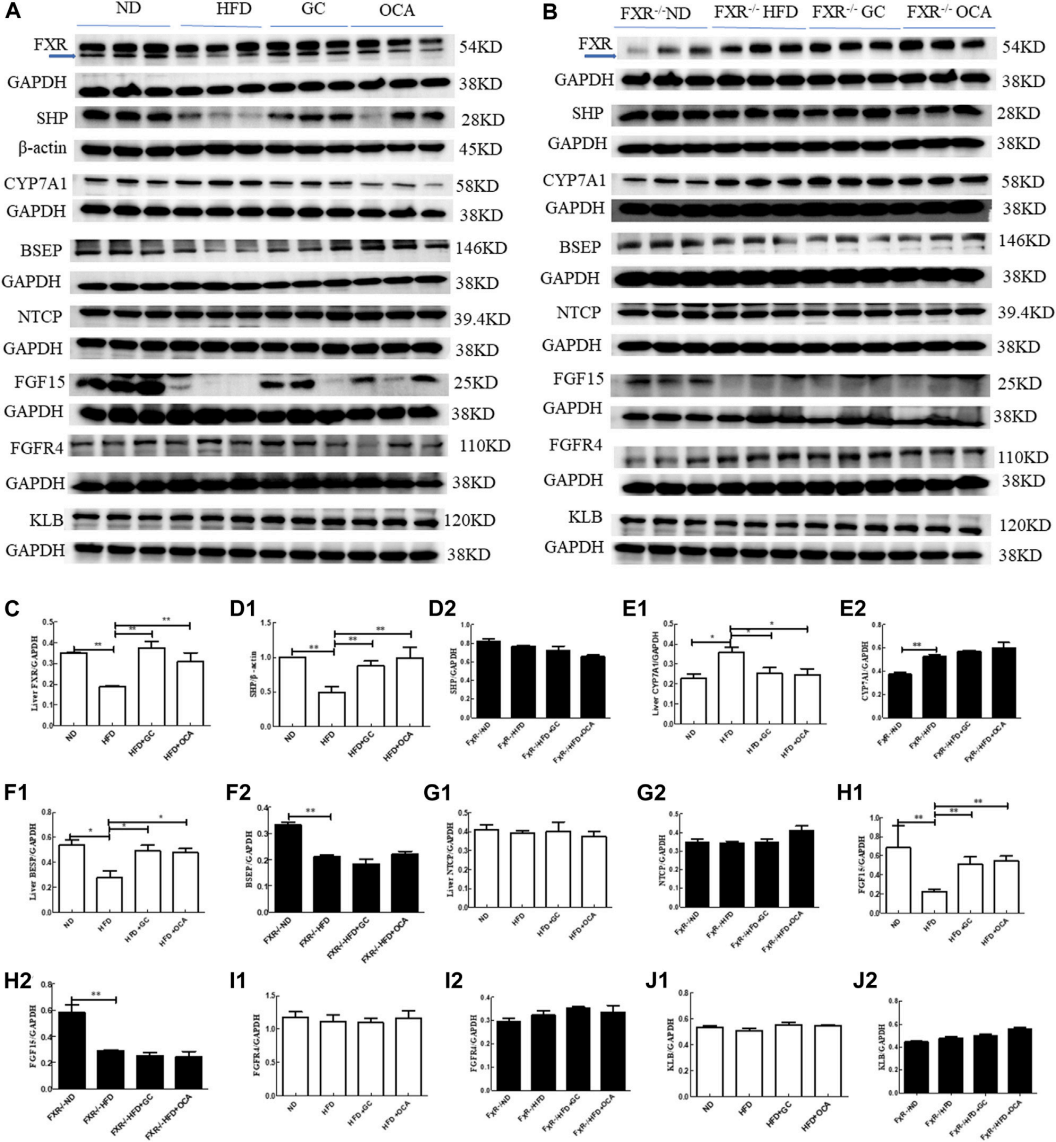
Changes in the liver tissue levels of FXR, SHP, CYP7A1, BSEP, FGFR4, NTCP, and KLB proteins and ileal tissue levels of FGF15 with intervention of GC or OCA in wild type and FXR^−/−^ mouse NAFLD models. **(A)** Western blot picture of wild-type mice. **(B)** Western blot picture of FXR^−/−^ mice. **(C)** Quantified data of FXR protein in liver tissues. **(D1, D2)** Quantified data of SHP protein in liver tissues. **(E1, E2)** Quantified data of CYP7A1 protein in liver tissues. **(F1, F2)** Quantified data of BSEP protein in liver tissues. **(G1, G2)** Quantified data of NTCP protein in liver tissues. **(H1, H2)** Quantified data of FGF15 protein in ileal tissues; **(I1, I2)** Quantified data of FGFR4 protein in liver tissues. **(J1, J2)** Quantified data of KLB protein in liver tissues. Abbreviations: NAFLD, non-alcoholic fatty liver disease; ND, normal diet; HFD, high-fat diet; GC, geniposide and chlorogenic acid combination treatment; OCA, obeticholic acid; FXR^−/−^, FXR knockout.*p < 0.05 and **p < 0.01 (*n* = 3 per group).

To explore the underlying molecular events of GC improving NASH in mice, we conducted experiments using *FXR*-knockout mice. First, we performed experiments on regular mice and found that compared to the ND mice, the levels of FXR, SHP, and BSEP proteins in the liver tissues and FGF15 protein in the ileal tissues were significantly reduced (*p* < 0.01 or *p* < 0.05), whereas the level of CYP7A1 was dramatically increased in the HFD mice (*p* < 0.05). In contrast, the levels of FXR, SHP, and BSEP proteins in the liver tissues and FGF15 protein in the ileal tissues were all increased (*p* < 0.01 or *p* < 0.05), whereas the level of CYP7A1 protein was reduced in the HFD + GC mice and the HFD + OCA mice vs. the HFD mice (*p* < 0.05, Figure 7).

However, compared to the FXR^−/−^ ND mice, the levels of SHP and BSEP proteins in the liver tissues and FGF15 protein in the ileal tissues were reduced (*p* < 0.01 or *p* < 0.05), whereas the level of CYP7A1 protein was increased in the FXR^−/−^ HFD mice (*p* < 0.05). However, the levels of SHP, BSEP, and CYP7A1 proteins in the liver tissues and FGF15 protein in the ileal tissues were not significantly different among the FXR^−/−^ HFD + GC, FXR^−/−^ HFD + OCA, and FXR^−/−^ HFD mice (*p* > 0.05; Figure 7).

## Discussion

In the current study, we evaluated the roles of geniposide and chlorogenic acid in alleviation of mouse NASH histology as well as the biochemical and other indicators *in vivo* in order to explore the underlying molecular events. We found that treatment with the combination of geniposide and chlorogenic acid was able to alleviate NASH in a mouse model *via* improvement of the gut microbiota and activation of FXR signaling and that its effect was truly better than each individual agent alone. Indeed, geniposide shows a variety of biological activities (Wang et al., 2017; Li et al., 2019), for example, geniposide has demonstrated hepatoprotective and cholagogic activities as well as anti-inflammatory and diabetic properties in animals and *in vitro* (Shan et al., 2017). In NAFLD, geniposide treatment has been reported to alleviate NAFLD (Shen et al., 2020), improve diabetic nephropathy (Dusabimana et al., 2021), and attenuate hyperglycemia-induced oxidative stress and inflammation (Tu et al., 2021). Moreover, although the medical value of chlorogenic acid usage in the clinic has not yet been established (Naveed et al., 2018; Miao and Xiang, 2020), it has shown biological activities in experimental animals and in cell lines. For example, chlorogenic acid has been demonstrated to be able to ease mouse ulcerative colitis induced by dextran sodium sulfate (Gao et al., 2019) and rat cardiovascular, liver, and metabolic changes induced by a high-carbohydrate diet and a HFD (Bhandarkar et al., 2019) as well as protect against metabolic syndrome (Santana-Gálvez et al., 2017). Taken together, it was hypothesized that if these two agents were used in combination, their biological activity would be increased *in vivo*. Our current study further supports this notion and found that their combination was better at attenuating NASH than each agent alone. However, further studies are needed to translate these findings into clinical usage.

Furthermore, the human gut microbiota have shown a role in regulating intestinal function and human health. A normal gut microbiota level is critical for maintaining immunologic, hormonal, and metabolic homeostasis of the human body. Therefore, any disorders might result in unwanted or excessive growth of pathogenic microorganisms that increase the levels of their related metabolites in the gut, which eventually get into the bloodstream and cause human diseases such as obesity (Korpela et al., 2016), diabetes (Muñoz-Garach et al., 2016), and inflammatory bowel disease (Sokol et al., 2017). In 2012, the US National Institutes of Health Human Microbiome Project issued the normal bacterial structure in the human body and showed that almost every individual routinely carries pathogens or microorganisms that may cause diseases; however, in healthy individuals, these pathogens may not cause disease because they just reside in the host body with the rest of the human microbiome (Abraham, 2012). Changes in the human microbiome will lead to disease development. Most recently, an abnormal gut microbiota has been reported to be associated with the development and progression of NAFLD (Khan et al., 2021). For example, after receiving a fecal microbiota transplantation (FMT) from NAFLD patients, the animals with a high-fat and high-sugar diet had a significant increase in mouse body weight and liver TG levels compared to those of mice receiving FMT from healthy controls, suggesting that the gut microbiota do participate in the development of obesity and hepatic steatosis (Burz et al., 2021). Another study has demonstrated that patients with NAFLD-associated HCC possess unique microbiota characteristics that regulate the peripheral immune response, further suggesting that the gut microbiota have a role in human diseases (Behary et al., 2021). Mechanistically, destruction of the intestinal vascular barrier induced by gut microbiota disorders is considered as the key factor in NASH progression, whereas repair and maintenance of the intestinal vascular barrier are able to effectively reverse NASH (Mouries et al., 2019). Thus, oral prebiotics, exercise, or dietary adjustments can modify the gut microbiota. In addition, a normal microbiota structure has been reported to decrease the severity of NAFLD or even reverse it (Aron-Wisnewsky et al., 2020). Moreover, it has been shown that FMT is able to restore normal intestinal permeability in NAFLD patients (Craven et al., 2020). To date, the safety and potential treatment risks of using FMT still need to be thoroughly evaluated before its routine clinical application for the treatment of NAFLD (Gupta et al., 2021). Our current data reveal that GC treatment reduced the liver lipid level and improved serum biochemical indicators in NASH mice. Meanwhile, GC treatment increased the levels of *Bacteroides*, Verrucomicrobia, and Tenericutes but significantly decreased the abundance of Firmicutes, Proteobacteria, and Deferribacteres in the intestines of NASH mice. The increase in Bacteroidetes in NASH mice was very significant, and the abundance of intestinal Bacteroidetes in NASH mice after the depletion of gut bacteria was very low. Importantly, the depletion of gut bacteria by the combination of antibiotics completely reversed the effect of GC treatment on NASH. We speculate that Bacteroidetes may be a key bacterial group on which GC acts in the treatment of NASH. Studies have shown that an increase in *Bacteroides* can induce the bile salt hydrolase (BSH) activity to in turn deconjugate bile acids and increase the bile acid level to activate FXR (Ovadia et al., 2020). The increase in the *Bacteroides* genus, especially increases in *Bacteroides ovatus* and *Bacteroides vulgatus*, can lead to an increase in intestinal BSH activity, which in turn leads to the uncoupling of TβMCA and a decrease in TβMCA, an FXR inhibitor (Mullish et al., 2019; Song et al., 2019). Our study also showed that treatment with GC upregulated the levels of *Bacteroides*, including *B. ovatus* and *B. vulgatus*, but reduced the TβMCA level to in turn activate the FXR signaling pathway.

Accumulated evidence shows that the metabolites of the gut bacteria, like bile acids and short-chain fatty acids, can bind to the intestinal epithelium and immune cells to alter their functions (Kayama et al., 2020; Hosseinkhani et al., 2021) to indirectly affect NAFLD development and progression (Chen et al., 2019). These metabolites are also able to break the intestinal barrier and appear in the bloodstream to mediate the development and progression of NAFLD by binding to various nuclear receptors (FXR and pregnane X receptor) in liver cells (Ding et al., 2019). Indeed, liver cholesterol is converted into bile acids by CYP7A1 and exported into the bile by BSEP, and it subsequently flows into the intestine to further promote lipid absorption. Approximately 95% of the intestinal bile acids are reabsorbed in the terminal ileum and upper colon by the apical sodium-dependent bile acid transporter and then transported back to the liver through the portal vein *via* NTCP, where hepatocytes take up these bile acids and resecrete them into the bile as the enterohepatic bile acid circulation for bile acid metabolism and homeostasis (Trauner et al., 2017). Bile acids can exert biological effects by activating or inhibiting FXR activity (Fiorucci and Distrutti, 2019; Fu et al., 2019; Liu et al., 2020), and the latter is able to adjust the liver glucose and lipid levels and inflammatory response as an important therapeutic target for NAFLD (Kim et al., 2016). In our current study, we administered antibiotics to eliminate the gut bacteria in a mouse NASH model *in vivo* and evaluated the effects of GC treatment in an *FXR*-knockout mouse model to elucidate the underlying molecular mechanism of its control of NASH by influencing the gut microbiota and bile acid-related signaling. We found that GC treatment improved intestinal and serum bile acid metabolism and activated FXR signaling in NASH mice. These data support that gut-microbiota, bile acids, and FXR signaling pathways play a role in the control of NASH development and progression; however, future studies are warranted to further confirm our findings.

FXR is primarily expressed in liver and ileal tissues to regulate glucose and lipid metabolism. FXR functional abnormalities have been associated with the development of NAFLD; thus, targeting FXR could be a therapeutic strategy to control NAFLD (Fiorucci et al., 2020). FXR reduces fat absorption and fatty acid synthesis and is associated with liver lipid metabolism in NAFLD (Clifford et al., 2021). In addition, the selective FXR agonist cilofexor (previously known as GS-9674) has been demonstrated to reduce hepatic steatosis in NASH patients and to have a good safety profile, indicating its potential treatment value in the control of NASH (Patel et al., 2020). The alternative FXR agonist MET409 also has been shown to decrease the liver lipid level in NASH patients (Harrison et al., 2021). Our current data showed that GC treatment significantly upregulated the expression of FXR protein in the liver tissues of NASH mice, whereas knockout of the *FXR* gene blocked the effects of GC on NASH mice, further supporting that FXR does play a role in regulating the effects of GC as an anti-NASH agent *in vivo*.

## Conclusion

In conclusion, our current study revealed that GC treatment effectively attenuated NASH in mice and that this combination had a better efficacy than each individual agent. GC treatment regulated the gut microbiota, especially the abundance of *Bacteroides* spp., and then regulated bile acid metabolism, especially the FXR activity regulator such as TaMCA, TβMCA, LCA, CDCA. As a result, GC treatment induced FXR expression and activated FXR signaling. However, the effects of the GC treatment were completely reversed by depletion of gut bacteria, and its effects were also lost or reduced in *FXR*-knockout mice, indicating that regulation of the gut microbiota and bile acid signaling by GC is required for its anti-NASH activity *in vivo*. Nevertheless, future studies are needed to further confirm our current data as well as to investigate these signaling pathways in the development and progression of NASH.

## Data Availability

The datasets presented in this study can be found in online repositories. The names of the repository/repositories and accession number(s) can be found below: https://www.ncbi.nlm.nih.gov/bioproject/PRJNA937722.

## Glossary

G: Geniposide
C: chlorogenic acid
GC: combination of geniposide and chlorogenic acid
NAFLD: Non-alcoholic fatty liver disease
NASH: non-alcoholic steatohepatitis
HCC: hepatocellular carcinoma
FXR: Farnesoid X receptor
SHP: Small heterodimer partner
CYP7A1: Cholesterol 7-alpha hydroxy-lase
FGFR4: Fibroblast growth factor receptor 4
BSEP: Bile salt export protein
NTCP: Na+-taurocholate cotransporting polypeptide
KLB: Klotho beta
FGF15: Fibroblast growth factor 15
HFD: High-fat diet
OCA: Obeticholic acid
ADW: antibiotics in drinking water
ALT: Alanine aminotransferase
AST: Aspartate aminotransferase
FINS: Fasting insulin
FBG: Fasting blood glucose
HOMA-IR: Homeostatic Model Assessment for Insulin Resistance
TG: Triglycerides
TC: Total cholesterol
LDL-C: Low density lipoprotein cholesterol
HDL-C: High density lipoprotein cholesterol
TBA: Total bile acid
CA: Cholic acid
TCA: Taurocholic acid
GCA: Glycocholic acid
TCDCA: Taurochenodeoxycholic acid
ACA: allo-cholic acid
DCA: deoxycholic acid
βCDCA: 3β-chenodeoxycholic acid
LCA: lithocholic acid
TαMCA: Tauro α-muricholic acid
TβMCA: Tauro β-muricholic acid
FMT: fecal microbiota transplantation.
