# Correction: Geniposide plus chlorogenic acid reverses non-alcoholic steatohepatitis via regulation of gut microbiota and bile acid signaling in a mouse model *in vivo*

**DOI:** 10.3389/fphar.2025.1657343

**Published:** 2025-12-01

**Authors:** Hongshan Li, Yingfei Xi, Xin Xin, Qin Feng, Yiyang Hu

**Affiliations:** 1 Institute of Liver Disease, Shuguang Hospital, Shanghai University of Traditional Chinese Medicine, Shanghai, China; 2 Liver Disease Department of Integrative Medicine, Ningbo No. 2 Hospital, Ningbo, Zhejiang, China; 3 Endocrine Department, Ningbo No. 2 Hospital, Ningbo, Zhejiang, China

**Keywords:** NASH, microbiota, bile acid, FXR, geniposide, chlorogenic acid

In the Funding statement, an incorrect number was provided for National Natural Science Foundation of China (#82174189 and #81873109). The correct number is National Natural Science Foundation of China (#82174186 and #81873109).

The original article has been updated.

